# Tight Shoes

**Published:** 1866-12

**Authors:** 


TIGHT SHOES

Interfere with the pleasure of locomotion, cause corns, and even rheumatic gout; hence it is worth while to repeat what we have formerly recommended as an infallible and easy method of having a new foot-covering fit as easily as an old shoe—just put on two pairs of thick stockings before the measure is taken or before fitting your feet with ready-made shoes; then when you get home pull off both pair, put on one thin pair, wear them for a few days and then put on thicker. This simple expedient will prevent an incalculable amount of discomfort, irritation and loss, in one year.

